# The importance of colonization pressure in multiresistant *Acinetobacter baumannii *acquisition in a Greek intensive care unit

**DOI:** 10.1186/cc11383

**Published:** 2012-06-13

**Authors:** Kostoula Arvaniti, Dimitrios Lathyris, Raymond Ruimy, Anna-Bettina Haidich, Vasiliki Koulourida, Pavlos Nikolaidis, Dimitrios Matamis, Spiros Miyakis

**Affiliations:** 1 Intensive Care Unit, "Papageorgiou" General Hospital, Periferiaki Odos, Thessaloniki, 56403, Greece; 2 Intensive Care Unit, "Gennimatas" General Hospital, Ethnikis Aminas 41, Thessaloniki, 54635, Greece; 3 Department of Clinical Microbiology, Centre Hospitalier Universitaire Bichat Claude-Bernard, Henri Huchard 46, Paris, 75018, France; 4 Department of Hygiene and Epidemiology, Medical School, Aristotelian University of Thessaloniki, 54124, Greece; 5 Department of Microbiology, "Papageorgiou" General Hospital, Periferiaki Odos, Thessaloniki, 56403, Greece; 6 1st Department of Internal Medicine, Medical School, Aristotle University of Thessaloniki, Stilponos Kyriakidi 1, 54636, Greece; 7 3rd Department of Internal Medicine, Medical School, Aristotle University of Thessaloniki, Periferiaki Odos, 56403, Greece

## Abstract

**Introduction:**

We investigated the role of colonization pressure on multiresistant *Acinetobacter baumannii *acquisition and defined patient-related predictors for carriage at admission and acquisition during hospitalization in intensive care unit (ICU) patients.

**Methods:**

This was a 12-month, prospective, cohort study of all patients admitted to a single ICU of a tertiary hospital. Screening samples were collected at ICU admission to identify imported carriers, and weekly during hospitalization to identify acquisition. Colonization pressure (carriers' patient-days × 100/all patients' patient-days) and the absolute number of carriers were calculated weekly, and the statistical correlation between these parameters and acquisition was explored. Multivariable analysis was performed to identify predictors for *A. baumannii *carriage at admission and acquisition during hospitalization. *A. baumannii *isolates were genotyped by repetitive-extragenic-palindromic polymerase chain reaction (PCR; rep-PCR).

**Results:**

At ICU admission, 284 patients were screened for carriage. *A. baumannii *was imported in 16 patients (5.6%), and acquisition occurred in 32 patients (15.7%). Acquisition was significantly correlated to weekly colonization pressure (correlation coefficient, 0.379; *P *= 0.004) and to the number of carriers per week (correlation coefficient, 0.499; *P <*0.001). More than one carrier per week significantly increased acquisition risk (two to three carriers, odds ratio (OR), 12.66; *P = *0.028; more than four carriers, OR, 25.33; *P *= 0.004). Predictors of carriage at admission were infection at admission (OR, 11.03; confidence interval (CI), 3.56 to 34.18; *P *< 0.01) and hospitalization days before ICU (OR, 1.09; CI, 1.01 to 1.16; *P *= 0.02). Predictors of acquisition were a medical reason for ICU admission (OR, 5.11; CI, 1.31 to 19.93; *P *= 0.02), duration of antibiotic administration in the unit (OR, 1.24; CI, 1.12 to 1.38; *P *< 0.001), and duration of mechanical ventilation (OR, 1.08; CI, 1.04 to 1.13; *P *= 0.001). All strains were multiresistant. Rep-PCR analysis showed one dominant cluster.

**Conclusions:**

Acquisition of multiresistant *A. baumannii *in ICU patients is strongly correlated to colonization pressure. High levels of colonization pressure and more than two carriers per week independently increase acquisition risk. Patient-related factors, such as infection at admission and long hospitalization before the ICU, can identify imported *A. baumannii *carriers. Medical patients with extended administration of antibiotics and long duration of mechanical ventilation in the ICU were the most vulnerable to acquisition.

## Introduction

*Acinetobacter baumannii *has become a troublesome emerging pathogen because of its wide range of resistance determinants and its environmental resilience [1,2]. It is responsible for severe nosocomial infections in intensive care unit (ICU) patients, mainly ventilator-associated pneumonia (VAP), bacteremia, and central nervous system infections [1,3-6]. Most of *A. baumannii *strains are resistant to major antimicrobial drugs (multidrug-resistant (MDRs)) [1,7,8]. MDR strains have become endemic in several health care environments and often cause sustained outbreaks [1,9-12]. Infected and colonized patients (carriers) represent major reservoirs for horizontal transmission and spread in nosocomial environment, through the hands of healthcare personnel [1,11-15].

Acquisition of MDR bacteria in an ICU depends on ICU-related variables (nurse-to-patient ratios, compliance with hand hygiene) and patient-related factors (severity of illness, prior hospitalization, invasive procedures, antibiotic use) [13-18]. Colonization pressure created by carriers (or colonized patients) has been indicated as a contributing factor for acquisition of MDR bacteria (methicillin-resistant *Staphylococcus aureus *(MRSA) and vancomycin-resistant *Enterococcus *sp. (VRE)) [19,20], but not yet extensively for *A. baumannii *[17,21].

Our hypothesis was that the acquisition of multiresistant *A. baumannii *in the ICU setting is strongly related to high levels of colonization pressure and to patient-related factors. To determine the role of these factors, we studied the effect of colonization pressure, nurse-to-patient ratios, severity, duration of invasive procedures, and use of antibiotics on *A. baumannii *acquisition. Additionally, we searched for predictors of carriage at ICU admission, such as comorbidities, severity and type of illness, prior hospitalization, and antibiotic use, assuming that they can help physicians promptly identify imported reservoirs of the pathogen.

## Materials and methods

The study was conducted in a general (medical, surgical, and trauma) adult, 15-bed ICU of a 750-bed, tertiary-care, university-affiliated hospital in Thessaloniki, Greece. The ICU had two subunits, one with 10 beds and another with five beds. Subunits had only two-bed rooms. Each nurse was responsible for two to three patients and had 8-hour shifts. Six physicians and five physicians-in-training worked in the unit every day.

Standard hygiene precautions [22] were applied. Hand antisepsis with alcohol hand rubs, before and after each contact with a patient and the surrounding surfaces, was implemented. Screening sampling at ICU admission and on a weekly basis was performed for *A. baumannii *and MRSA. Contact isolation precautions with protective gowns and gloves were applied for proven cases of carriage or infection with *A. baumannii *or MRSA, as well as for patients infected with carbapenem-resistant *Pseudomonas aeruginosa *or *Klebsiella pneumoniae *[23]. For all those patients, confinement (isolation) was not performed, because the unit was composed of two-bed rooms, whereas nurse cohorting was implemented when daily nurse-to-patient ratios were below or equal to 1:2. For patients transferred from other institutions or other ICUs, contact precautions were applied until results of screening samples were collected at ICU admission. Chlorhexidine was used for daily body washing of patients.

Hand-hygiene compliance was not monitored. Educational meetings were being held every 3 months to emphasize the importance of hand hygiene and contact precautions to ICU staff and to provide essential feedback. Infection-control policy included continuous surveillance of nosocomial infections and was revised yearly by an ICU physician specialized in infectious diseases and the Institute's Infection Control Committee. Nosocomial infections in the ICU (that is, infections occurring after the first 48 hours in the unit) were evaluated according to CDC/NHSN definitions [24,25]. Antibiotic treatment was based on epidemiologic data and guidelines for each type of infection [26-30]. No selective digestive decontamination or antibiotic rotation was performed. Deescalation was routinely effectuated after culture results [31,32]. The most common empiric antibiotic regimen in case of clinical suspicion of severe nosocomial sepsis was imipenem for the coverage of *A. baumannii *and *P. aeruginosa*. An antibiotic with activity against MRSA was initially prescribed in case of septic shock, presence of a prosthetic endovascular or an orthopedic device, transfer from wards with a high prevalence of MRSA, or hospitalization in the ICU of more than one patient with MRSA-positive samples (screening or clinical samples).

This was a prospective cohort study that included all consecutive patients admitted into the unit between August 2008 and July 2009. Only the first ICU admission per patient was included in the analysis. Patients were evaluated for (a) carriage at admission and (b) acquisition during hospitalization. Patients without screening samples at admission were excluded. Imported carriers and those without follow-up samples (due to discharge or death before the scheduled follow-up sampling) were excluded from acquisition analysis. Only the initial isolate from each patient was counted and underwent molecular typing.

The study was approved by the Institute's Ethics committee. No written consent was obtained from patients, because no therapeutic intervention was undertaken; carrier screening was part of the routine practice in the unit; and all data were anonymous.

### Data collection

Demographic data, underlying diseases, severity scores, invasive procedures, antibiotic use, weekly nurse-to-patient ratios, and weekly levels of colonization pressure were collected prospectively by using a standard written questionnaire. The accuracy and completion of each questionnaire was evaluated every week by two participating authors.

More specifically, the following data were collected: age, sex, date of hospital and ICU admission, diagnosis on ICU admission, comorbidities, McCabe score, immunodeficiency (hematologic malignancy, metastatic cancer, AIDS, immunosuppressive therapy or chemotherapy, corticosteroids (daily dose ≥ 1 mg/kg of prednisolone for 1 month or any daily dose for > 1 month), neutropenia), Acute Physiology and Chronic Health Evaluation-APACHE II, Simplified Acute Physiologic Score-SAPS II, and Organ Dysfunction, and/or Infection-ODIN on ICU admission, length of ICU stay, and ICU outcome (death or not)) [33-36]. Imported and acquired cases and time to *A. baumannii *acquisition were recorded. During ICU hospitalization, duration of antibiotic administration, of invasive procedures (mechanical ventilation (MV), central venous (CVC) and arterial catheterization, enteral feeding tube, urinary tract catheterization) and duration of propofol infusion were recorded in the questionnaire.

The unit of time chosen for measurements was the week (from Monday through Sunday). Nurse-to-patient ratios (N/P) per week were monitored, and weekly colonization pressure created by all (imported and acquired) *A. baumannii *carriers was calculated, as described by Merrer *et al*. [20]:

$$Colonization\;pressure=\frac{\text{number of (admitted + acquired)carriers' patient - days in the week before acquisition}\times \text{100}}{\text{total number of patient - days in the week before acquisition}}$$

Samples for carriage were collected with cotton swabs (Transwab for aerobic and anaerobic; Medical Wire and Equipment Ltd., Corsham, England) from pharynx, skin, and rectum (screening samples), at ICU admission (initial sample, that is, within the first 48 hours of admission) and afterward on a weekly basis (every Monday), (follow-up samples) until discharge or death. Swabs were processed in specific media, as previously described [37]. Identification, confirmation, and susceptibility testing were performed with the Wider automated system, according to the National Committee for Clinical Laboratory Standards (NCCLS) criteria and breakpoints [38]. Microbiologic data including cultures and antibiotic susceptibility tests of screening samples were interpreted by a senior microbiologist on a daily schedule. All isolates were kept at -35°C for molecular analysis, which was effectuated and interpreted blindly by a senior specialized microbiologist, after completion of all other data collection. Bacteria from the frozen stock were inoculated onto Muller-Hinton agar (Sanofi-Pasteur, Marne la Coquette, France). PCR amplification of repetitive extragenic palindromic sequences (Rep-PCR) was performed for unique isolates by using the primers and the protocol described by Snelling *et al*. [39].

### Definitions

A patient was considered a carrier if he or she had at least one positive screening sample. Carriage at ICU admission (imported carriage) and acquisition during hospitalization in the unit (acquired carriage) were defined as isolation of *A. baumannii *from at least one screening sample obtained within or later than the first 48 hours of admission, respectively. Imported carriers were considered to be carriers throughout their entire stay, and acquired carriers were considered to be carriers from the date of the first positive sample until discharge or death. An infection was considered to be present at ICU admission if it was diagnosed within the first 48 hours in the unit. Multiresistance was defined as resistance to more than two of the following five drug classes: antipseudomonal cephalosporins (ceftazidime or cefepime), antipseudomonal carbapenems (imipenem or meropenem), ampicillin-sulbactam, fluoroquinolones (ciprofloxacin or levofloxacin), and aminoglycosides (gentamicin, tobramycin, or amikacin) [1].

### Statistical analyses

Descriptive analysis of the data was performed. Continuous variables were presented as median (interquartile range [IQR]) values, as they were not normally distributed (if not, mean ± SD was added), and categoric variables, as counts and percentages. Logistic regression analyses were performed to assess predictors of *A. baumannii *carriage at (a) admission and (b) acquisition during hospitalization. All variables with a *P *value less than 0.05 in univariable analysis were included in a logistic regression model for multivariable analysis. To preserve the sufficient events per variable, from the significant univariable parameters, all possible two-variable models were determined for imported *A. baumannii *carriers (*n *= 16) and all possible three-variable models were determined for acquired carriers (*n *= 32). The selection of the final multivariable model presented was the one with the better fit in terms of deviance, the one with the smallest -2-log likelihood. Also, the goodness of fit was evaluated with the Hosmer-Lemeshow test, in which an insignificant result indicated a good fit [40]. Predictor variables that were highly correlated were not entered together in the model to preserve for collinearity problems. Odds ratios were presented with the corresponding 95% confidence intervals (OR, CI 95%). Correlation between acquisition and weekly values of parameters (colonization pressure, total *A. baumannii *carriers, and nurse-to-patient ratio) was calculated by using the Pearson correlation coefficient. OR and 95% CI were used to evaluate the risk for acquisition at different numbers of total *A. baumannii *carriers hospitalized per week in the ICU.

The Rep-PCR patterns were analyzed by using the Bionumerics software (V1.5; Applied Maths, Ghent, Belgium) in a blind fashion (that is, without knowledge of the clinical data). Distance matrices were determined by using the Pearson correlation coefficient, and dendrograms were created by using the unweighted pair group method with arithmetic averages (UPGMA).

All tests were two-sided, and a *P *value less than 0.05 was considered statistically significant in the multivariable analyses. The analyses were conducted by using PASW Statistics (version 18; SPSS, Chicago, IL, USA).

## Results

During the study period, 301 patients were admitted in the ICU and assessed for eligibility. Forty-five (15%) patients had an infection at ICU admission; 120 (40%) had received previously antibiotics, and 168 (56%) were hospitalized in the preceding 12 months. For 185 (61%) patients, the main reason for ICU admission was medical; 126 (42%) patients were transferred from the emergency department; 143 (50%) from other hospital wards; and 15 (5%) from ICUs of other institutions. Seventy-six (25%) patients died in the ICU.

Seventeen (5.6%) patients 301 were excluded from analysis for *A. baumannii *carrier status because sampling was not performed (four died in less than 2 hours; for eight patients, the sampling was neglected, and five patients were readmitted). Finally, 284 (94.3%) of 301 patients were evaluated for *A. baumannii *carriage (screened population).

Sixteen (5.6%) of 284 patients were imported carriers at ICU admission and were excluded from acquisition analysis. Two hundred sixty-eight (94.4%) of 284 patients were evaluated for *A. baumannii *acquisition. Of these, 64 (23.9%) patients were excluded, as they had no follow-up sample (48 were discharged, and 16 died before the scheduled sampling).

**Figure 1:**
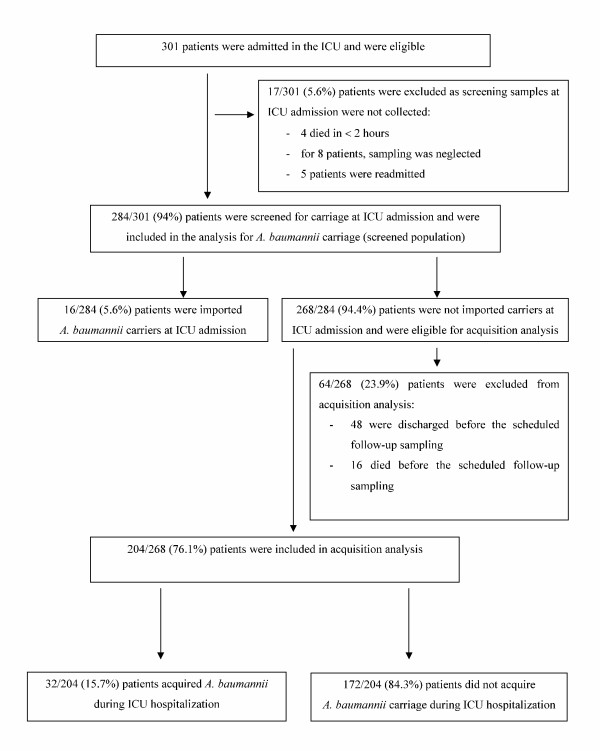
**Flow chart of the patients**.

Finally, 204 (76%) patients were evaluated for *A. baumannii *acquisition. Thirty-two (15.7%) of 204 patients acquired *A. baumannii *in the ICU after a delay of a median 12 days (IQR, 10 to 17.5) (Figure 1).

The pharynx was the most frequent isolation site for both imported (11 of 16, 69%) and acquired (19 of 32, 59%) carriers. Skin carriage was identified in four (25%) of 16 imported and nine (28%) of 32 acquired carriers, whereas the rectum was found to be positive in only one (6%) of 16 imported and four (13%) of 32 acquired carriers.

The median calculated weekly colonization pressure was 25.6% (IQR, 9.3 to 47.8) and ranged from 0 to 96%. During 28 (54% of the study period) of 52 study weeks, colonization pressure exceeded the median value. The median number of all *A. baumannii *carriers per week was 3.5 (IQR, 1 to7) and during 30 (58% of the study period) of 52 study weeks, more than 3.5 carriers were concomitantly hospitalized in the unit. The mean value (± SD) of the weekly nurse-to-patient ratio was 1:2.7 (± 0.3) (range, 1:1.8 to 1:3.3) and median 1:2.7 (IQR, 1:2.5 to 1:2.7), whereas, for 19 (36.5% of the study period) of 52 weeks, ratios were below these values. Weekly colonization pressure (correlation coefficient, 0.379; *P *= 0.004) and total number of *A. baumannii *carriers per week (correlation coefficient, 0.499; *P *= 0.0001) were significantly associated with *A. baumannii *acquisition, but not the nurse-to-patient ratio (correlation coefficient, -0.064; *P *= 0.064). The presence of more than one *A. baumannii *carrier in the unit per week, either imported or acquired, strongly predicted increased acquisition risk (two to three carriers per week, OR, 12.66; *P = *0.028; and more than four carriers, OR, 25.33; *P *= 0.004).

ODIN score, infection at ICU admission, hospitalization before ICU admission, hospital days before ICU admission, and antibiotic administration before ICU admission were identified as risk factors for imported carriage in univariable analysis (Table 1). A medical reason for ICU admission, ICU days before acquisition, antibiotic and propofol administration in the unit, days of antibiotics and propofol use in the unit, and duration of invasive procedures (mechanical ventilation, enteral tube feeding, CVC, arterial and urinary tract catheterization) were associated with acquisition in univariable analysis (Table 2).

**Table 1:** Univariable analysis of risk factors for multiresistant *Acetinobacter baumannii *carriage at ICU admission

Variable			Univariable analysis	
	***A. baumannii *carriers at ICU admission (*n *= 16)**	**Noncarriers at ICU admission (*n *= 268)**	**OR (95% CI)**	***P *value**

Age, median [IQR]	67.5 [38-73.5]	64 [41-72]	1 (0.98-1.03)	0.77
Males *n *(%)	13 (81)	173 (65)	2.38 (0.66-8.56)	0.18
APACHE II, median [IQR]	21 [15.1-26]	17 [13-23]	1.04 (0.98-1.09)	0.19
SAPS II, median [IQR]	50.5 [40-64.2]	41 [28-54]	1.02 (0.99-1.04)	0.15
ODIN, median [IQR]	3 [2.25-4]	2 [1-3]	1.50 (1.04-2.15)	0.03
McCabe *n *(%)				
0-1	9 (56)	173 (59.7)	0.70 (0.26-1.95)	0.50
2-3	7 (44)	95 (32.8)		
Reason for ICU admission *n *(%)				
Medical reason	11 (68.7)	174 (64.9)	1.189 (0.4-3.52)	0.75
Surgery before ICU admission	5 (31)	94 (36)		
Trauma	2 (12.5)	51 (19)	0.60 (0.13-2.76)	0.52
Infection at ICU admission	10 (63)	37 (14)	10.41 (3.57-30.34)	0.001
Main reason for ICU admission *n *(%)				
Other	3 (18.75)	68 (25.4)	1	
Acute respiratory failure	6 (38)	64 (24)	1.32 (0.34-5.10)	0.69
Neurologic disorder	6 (38)	107 (40)	0.94 (0.26-3.42)	0.93
Hemodynamic disorder	1 (6.25)	29 (11)	0.58 (0.06-5.35)	0.63
Hospitalization before ICU admission *n *(%)	14 (87.6)	144 (53.7)	5.18 (1.15-23.2)	0.03
In ward	11/14 (78.5)	132/144 (91.7)		
In another ICU	3/14 (21.4)	12/144 (8.3)		
Hospital days before ICU admission, median [IQR]	5 [2.25-12.25]	0 [0-4]	1.08 (1.02-1.13)	0.005
Antibiotic use before ICU admission *n *(%)	15 (94)	105 (39)	23.29 (3.03-178.7)	0.002

APACHE II, Acute Physiologic And Chronic Health Evaluation; CVC, central venous catheter; ICU, intensive care unit; IQR, interquartile range; ODIN, Organ Dysfunctions and/or Infection; OR, odds ratio; Other, postsurgery follow-up, trauma, cancer, and immunodeficiency; SAPS II, Simplified Acute Physiology Score II.

**Table 2:** Univariable analysis of risk factors for multiresistant *Acetinobacter baumannii *acquisition during ICU hospitalization

Variable			Univariable analysis	
	**Patients with *A. Baumannii *acquisition (*n *= 32)**	**Patients without *A. baumannii *acquisition (*n *= 172)**	**OR (95% CI)**	***P *value**

Age, median [IQR]	51 (32-69)	63 (40.25-72)	0.98 (0.97-1.003)	0.10
Males *n *(%)	24 (75)	108 (63)	1.78 (0.75-4.19)	0.19
APACHE II, median [IQR]	17 (13-22)	17 (13-22.75)	0.98 (0.93-1.03)	0.39
SAPS II, median [IQR]	40 (27.2-52)	41 (29-53)	0.99 (0.97-1.01)	0.31
ODIN, median [IQR]	2 (1.25-3)	2 (1-3)	1.03 (0.76-1.38)	0.86
Mc Cabe *n *(%)				
0-1	25 (75)	109 (63)	2.06 (0.85-5.05)	0.11
2-3	7 (25)	63 (37)		
Reason for ICU admission *n *(%)				
Medical reason for ICU admission	28 (88)	110 (64)	3.85 (1.29-11.48)	0.016
Surgery before ICU admission	4 (12)	62 (36)		
Trauma	11 (34)	33 (19)	2.2 (0.97-5.02)	0.06
Infection at ICU admission	6 (19)	23 (20)	1.49 (0.57-4.02)	0.43
Main reason for ICU admission *n *(%)				
Other	6 (18.7)	42 (24.4)		
Acute respiratory failure	9 (28)	46 (27)	0.73 (0.24-2.22)	0.58
Neurologic disorder	15 (47)	66 (38)	0.63 (0.22-1.75)	0.37
Hemodynamic disorder	2 (6)	18 (15)	1.28 (0.23-6.99)	0.77
ICU days before acquisition, median [IQR]	11.5 (9-18.75)	5 (3-10)	1.03 (1.01-1.06)	0.025
Antibiotic use before acquisition *n *(%)	32 (100)	127 (74)	10.98 (1.46-82.8)	0.02
Days of antibiotics median [IQR]	7 (5-9.75)	3 (0-5)	1.28 (1.15-1.42)	< 0.001
Mechanical ventilation before acquisition *n *(%)	32 (100)	152 (88)	4.08 (0.53-31.5)	0.18
Days of mechanical ventilation, median [IQR]	11.5 (8.25-16)	4 (2-8)	1.09 (1.05-1.14)	< 0.001
CVC in the ICU before acquisition *n *(%)	26 (81)	152 (88)	0.57 (0.209-1.554)	0.27
Days of CVC, median [IQR]	12 (9-15.7)	6 (3-10)	1.07 (1.03-1.12)	0.001
Propofol use before acquisition *n *(%)	12 (38)	131 (76)	0.19 (0.09-0.42)	< 0.001
Days of propofol, median [IQR]	5 (3.75-7)	2 (1-3)	1.20 (1.07-1.36)	0.003
Arterial catheter before acquisition *n *(%)	20 (63)	154 (89.5)	2.06 (0.95-4.69)	0.07
Days of arterial catheter, median [IQR]	11 (8-14.7)	6 (3-9)	1.07 (1.02-1.13)	0.003
Enteral feeding catheter before acquisition *n *(%)	31 (97)	155 (90)	1.48 (0.64-3.39)	0.36
Days of enteral feeding tube, median [IQR]	12 (9.2-19)	4 (3-9)	1.07 (1.04-1.10)	< 0.001
Urinary catheter before acquisition *n *(%)	32 (100)	165 (96)	2.53 (0.32-20.08)	0.38
Days of urinary tract catheterization, median [IQR]	12.5 (9-18.25)	6 (3-11)	1.07 (1.04-1.10)	< 0.001

APACHE II, Acute Physiologic And Chronic Health Evaluation; CI, confidence interval; CVC, central venous catheter; ICU, intensive care unit; IQR, interquartile range; ODIN, Organ Dysfunctions and/or Infection; OR, odds ratio; other, postsurgery follow-up, trauma, cancer, and immunodeficiency; SAPS II, Simplified Acute Physiology Score II.

Infection at ICU admission (OR, 11.03; 95% CI, 3.56 to 34.18; *P *< 0.01) and duration of prior hospitalization were independently associated with carriage at admission in multivariable analysis (OR, 1.09; 95% CI, 1.01 to 1.16; *P *= 0.02) (Table 3). Hospitalization before ICU admission and hospital days before ICU were not entered together in the logistic regression models because of collinearity.

**Table 3:** Multivariable analysis for multiresistant *Acetinobacter baumannii *carriage at ICU admission and acquisition during hospitalization

Variable	Multivariable analysis for *A. baumannii *carriage at ICU admission^a^		Multivariable analysis for *A. baumannii *acquisition^bc^	
	**OR (95% CI)**	***P *value**	**OR (95% CI)**	***P *value**
	
Infection at ICU admission	11.03 (3.56-34.18)	< 0.01		
Days in hospital before ICU admission	1.09 (1.01-1.16)	0.02		
				
Medical reason for ICU admission			5.11 (1.31-19.93)	0.02
Days of antibiotics before acquisition			1.24 (1.12-1.38)	< 0.01
Days of mechanical ventilation before acquisition			1.08 (1.04-1.13)	< 0.01

**^a^**The best two-variable model with deviance 92.9 and Hosmer-Lemeshow test, χ^2 ^= 0.92; *P *= 0.1. **^b^**The best three-variable model with deviance 132.3 and Hosmer-Lemeshow test, χ^2 ^= 11.62; *P *= 0.17. **^c^**Factors not analyzed in the multivariable analysis for multiresistant *A. baumannii *acquisition: (a) duration of enteral feeding tube, of arterial and urinary tract catheterization during hospitalization due to collinearity with duration of mechanical ventilation, and (b) antibiotic use and mechanical ventilation before acquisition, as they were found in all patients with acquisition. Propofol administration was not analyzed in the multivariable analysis for acquisition, as categoric variable, because of clinical criteria. CI, confidence interval; CVC, central venous catheter; ICU, intensive care unit; ODIN, Organ Dysfunctions and/or Infection; OR, odds ratio.

In multivariable analysis for the acquisition, medical reason for ICU admission (OR, 5.11; CI, 1.31 to 19.93; *P *= 0.02), antibiotic days in the ICU (OR, 1.24; CI, 1.12 to 1.38; *P *< 0.001), and mechanical ventilation days (OR, 1.08; CI, 1.04 to 1.13; *P *= 0.001) were found to be significant risk factors (Table 3). The following factors were not analyzed in the multivariable analysis for acquisition: duration of enteral tube feeding, and of arterial and urinary tract catheterization due to collinearity with duration of mechanical ventilation. Antibiotic use and mechanical ventilation before acquisition were excluded, as they were found in all acquired carriers. Propofol use, as a categoric variable, was not analyzed in the acquisition multivariable analysis because of clinical criteria. Although it was clearly used in fewer acquired carriers than in nonacquired ones, we considered that this result was found by chance, as our study was not randomized, and propofol cannot be considered a protective factor (OR, 0.188; CI, 0.085 to 0.417; *P *< 0.001) for multiresistant *A. baumannii *acquisition. Duration of mechanical ventilation and of CVC catheterization was not analyzed in the same logistic regression model because of collinearity. When mechanical ventilation days were replaced in the regression model by CVC days, similar results were obtained, but the model that contained the duration of mechanical ventilation had a better fit (-2 log likelihood, 132.4), than did the one with duration of CVC catheterization (-2 log likelihood, 140.4).

### Microbiologic results

**Figure 2:**
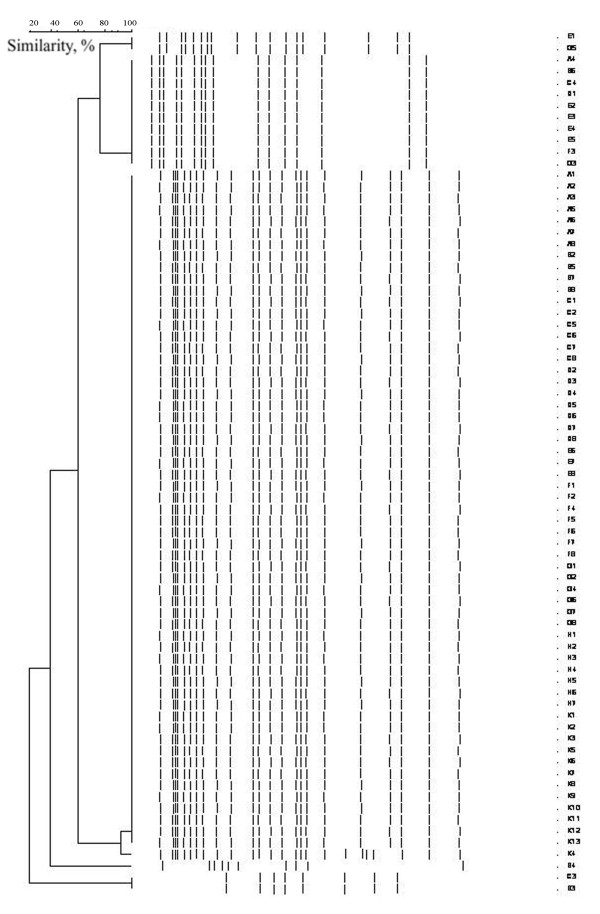
**Dendrogram illustrating genetic diversity among multiresistant *A. baumannii *strains**. Molecular analysis revealed one predominant cluster (III) of *A. baumannii *isolates among other minor ones (I, II, V). Cluster III included study isolates A_1-2-3-5-6_, B_2-5-7_, C_1-2-5-6-7_, D_2-3-4-5-6_, E_6-7-8_, F_1-2-4-5-6_, and G_1-2-4-6-7-8_. Ten (62.5%) of 16 imported strains and 23 (72%) of 32 acquired strains belonged to cluster III.

All isolates were multiresistant (Table 4). Nonsusceptibility to imipenem was noted in 6 (37.5%) of 16 imported and 15 (46.8%) of 32 acquired isolates. Nonsusceptibility to colistin was rarely found (6%), and this was exclusively among acquired isolates. High rates of nonsusceptibility to other antimicrobials were recorded. All recovered *A. baumannii *isolates were genotyped. Rep-PCR patterns revealed clusters of isolates (Figure 2 dendrogram). Among imported strains, 10 (62.5%) of 16 belonged to cluster III, and six (37.5%) of 16 to clusters I, II, and V. Among the 32 acquired strains, 23 (72%) belonged to cluster III, and nine (28%), to cluster II.

**Table 4:** Nonsusceptibility of *Acetinobacter baumannii *isolates to antibiotics

Antibiotic	Imported *A. baumannii *isolates	Acquired *A. baumannii *isolates
	
	I+R %	I+R %
Piperacillin/tazobactam	81	94

Ceftazidime	94	91

Cefepime	94	88

Gentamicin	94	100

Amikacin	94	91

Tobramycin	88	100

Aztreonam	94	100

Ciprofloxacin	88	100

Imipenem	38	47

Meropenem	63	59

Cotrimoxazole	88	91

Colistin	0	6

I, intermediate; R, resistant.

## Discussion

This prospective cohort study, performed in a general ICU with severely ill patients, demonstrated the impact of colonization pressure and patient-related variables (medical reason for ICU admissions, antibiotic exposure, and invasive procedures) on multiresistant *A. baumannii *acquisition during ICU hospitalization and identified predictors of multiresistant *A. baumannii *carriage at ICU admission.

The present study is the first to correlate *A. baumannii *acquisition with colonization pressure, signifying the important role of this parameter in ICU setting. The study also showed that the absolute number of all (imported and acquired) multiresistant *A. baumannii *carriers hospitalized in an ICU during a specific period (a week), could be related to the increased likelihood for new *A. baumannii *acquisitions. Previous studies already presented similar results for VRE and MRSA [19,20], probably because of the same transmission mode, mainly via contacts with the hands of personnel. Two other trials supported the influence of a high density of *A. baumannii *infections on dissemination of the pathogen, especially during outbreaks of carbapenem-resistant strains [21,41]. For carbapenem-resistant *K. pneumoniae*, a direct relation between colonization pressure and nosocomial acquisition of the pathogen also was demonstrated [42].

Absence of correlation between acquisition and nurse-to-patient ratios in the current study certainly does not underestimate the influence of understaffing and overcrowding on multiresistant *A. baumannii *acquisition in ICUs, already shown by others [21,43,44]. However, in settings with a high incidence of MDR bacteria, colonization pressure could be considered more sensitive than staff ratios or patient-related variables and should be used to alert health care personnel of increased multiresistant *A. baumannii *acquisition risk [19,20].

Multiresistant *A. baumannii *carriers represented an essential reservoir of the pathogen in the unit. Imported carriers as initial sources and acquired carriers as secondary sources of the pathogen generated a constant colonization pressure that facilitated new acquisitions in the ICU. Each *A. baumannii *carrier considerably increased the acquisition risk for other patients. During the study period, cross-transmission probably occurred because of lapses in infection-control measures (hand hygiene, contact precautions) in repeated circumstances with high levels of colonization pressure. The architectural design of the unit (only two-bed rooms available) precluded patient isolation, which could possibly help reduce *A. baumannii *dissemination. High patient-severity scores, prior hospitalization, and prolonged exposure to invasive procedures and antibiotics increased, for each ICU patient that was previously exposed to high levels of colonization pressure, the probability of acquiring one of the circulating clusters.

Imported *A. baumannii *carriers were admitted in the unit from other hospital wards or other ICUs in town, demonstrating dissemination in the entire hospital and implying interinstitutional or even regional transmission of the pathogen [1,9-12].

In accordance with other reports, we found that duration of hospitalization before ICU admission was independently associated with imported carriage [16,45]. For each previous day in hospital, the risk of *A. baumannii *carriage at ICU admission increased by 8% (*P *= 0.034).

Prior antibiotic use and duration of antibiotic exposure had already been identified as predictors of acquisition of MDR bacteria containing specific resistance mechanisms [19,46,47]. Nevertheless, direct association between duration of antibiotic administration and multiresistant *A. baumannii *acquisition in the ICU, as shown in the study, was not previously recognized.

Medical patients and patients with longer duration of mechanical ventilation were at higher risk of acquiring multiresistant *A. baumannii *during their ICU stay. For medical patients, this could be attributed to underlying comorbidities; however, this hypothesis can be neither adopted nor rejected by the current study. Duration of mechanical ventilation or CVC catheterization has already been described as a risk factor for *A. baumannii *acquisition [48,49].

Molecular analysis revealed one predominant cluster of *A. baumannii *isolates among other minor ones, probably reflecting endemicity and cross-transmission [50].

The study had limitations. First, compliance with hand antisepsis and contact precautions was not monitored. However, as our study was a nonblind prospective one, the announcement of adherence monitoring could have influenced the staff attitude and possibly biased the results. Our aim was to evaluate *A. baumannii *acquisitions under the current infection-control practices in the ICU and not to estimate the level of staff compliance or the effectiveness of standard and contact precautions on the control of an outbreak.

Second, the workload for each ICU patient was not calculated. Instead, we evaluated an important number of patient-dependent factors, such as comorbidities, severity scores, duration of invasive procedures, and antibiotic exposure, which indirectly indicate the degree of nurse workloads.

## Conclusion

An important impact of colonization pressure on multiresistant *A. baumannii *acquisition among ICU patients was demonstrated, and this finding probably adds new perspectives to the causative approach to sustained endemic situations. ICUs with similar problems should apply carriage-surveillance practices and implement additional infection-control measures immediately after identification of the first two carriers, rather than expect manifestation of infections or further spread of the pathogen. The factor Time, either as hospital days before ICU admission, or as antibiotic treatment-days and duration of mechanical ventilation during the ICU stay, was recognized as an independent risk factor for imported and acquired carriage, respectively. In settings in which carriage screening is unattainable because of increased cost and workload, infection-control measures should focus on, at least, the timely checking of patients with these risk factors.

Our study suggests that colonization pressure and patient-related risk factors should be considered when multiresistant *A. baumannii *acquisition remains an unresolved issue in the ICU setting. Their consideration may trigger implementation of appropriate preventive measures such as better hand-hygiene compliance, reduction of health care worker movement between carriers and noncarriers, creation of cohorts of either patients or nursing staff, and limitation of the number of physicians entering patient rooms during rounds.

## Key messages

• Multiresistant *A. baumannii *acquisition is correlated to colonization pressure and the total number of carriers per week. In endemic ICUs, surveillance practices and additional infection-control measures should be implemented immediately after identification of the first two carriers, rather than expecting further dissemination of the pathogen or infection manifestation.

• An important proportion of multiresistant *A. baumannii *carriers are admitted into the ICU from other wards of the hospital or other hospitals, creating a substantial reservoir for new acquisitions in ICUs in which the pathogen is endemic.

• Duration of hospitalization before the ICU is an important predictor of multiresistant *A. baumannii *carriage at ICU admission.

• Durations of antibiotic administration and mechanical ventilation in the ICU are independent risk factors for multiresistant *A. baumannii *acquisition.

## Abbreviations

APACHE II: Acute Physiologic and Chronic Health Evaluation; CI: confidence interval; CVC: central venous catheter; ICU: intensive care unit; IQR: interquartile range; ODIN: Organ Dysfunctions and/or Infection; OR: odds ratio; SAPS II: Simplified Acute Physiology Score II.

## Competing interests

The authors declare that they have no competing interests.

## Authors' contributions

KA and DL conceived the study and elaborated the data-analysis plan; KA, DM, RR, PN, and VK were responsible for data acquisition; KA and DL revised study data; DL and ABH were responsible for statistical analysis; DL, ABH, KA, and RR performed data interpretation; KA and DL drafted the final manuscript; and KA and SM revised all presented data and reevaluated the manuscript. The corresponding author had full access to all the data in the study and had the final responsibility for the decision to submit for publication. All authors critically commented on the draft and approved the final version.
